# Comparative efficacy of different tenderizing agents and time on Physicochemical textural and organoleptic properties of squid (*Todarodes pacificus*) muscle

**DOI:** 10.1002/fsn3.3751

**Published:** 2023-10-13

**Authors:** Mehtap Baykal, Mehmet Çelik, Ladine Çelik, Aygül Küçükgülmez

**Affiliations:** ^1^ Vocational School of Yumurtalık, Tourism and Hotel Management, Cukurova University Adana Turkey; ^2^ Faculty of Ceyhan Veterinary Medicine, Cukurova University Adana Turkey; ^3^ Department of Animal Sciences, Agricultural Faculty Cukurova University Adana Turkey; ^4^ Department of Seafood Processing Technology Faculty of Fisheries, Cukurova University Adana Turkey

**Keywords:** marination, milk, mineral water, squid, tenderization, yeast

## Abstract

This study aimed to investigate the chemical, physical, textural, and sensory properties of the squid (*Todarodes pacificus*) muscle during different tenderization agents (yeast, milk, and mineral water) and times (3, 6, 12, and 24 h). The results of the analyses showed that different treatments and their durations affected the dry matter, ash, lipid, and crude protein content of the squid. According to sodium dodecyl sulfate–polyacrylamide gel electrophoresis results, it was observed that there was a slight decrease in band intensities based on different treatments and their durations. However, no significant changes were observed in myosin actin and paramyosin bands. It was found that the hardness (359.7 *N*), cohesiveness (0.63), and gumminess (233.2 *N*) parameters increased at the 6th hour, decreased at the 12th hour, and increased again at the 24th hour of the treatment. According to the scanning electron microscopy results, the most affected groups by the application and duration were found to be the mineral water group at the 12th and 24th hours, and the milk group at the 24th hour. Fibers in all marinated squid muscles were observed to spring significantly more compared to the positive and negative control groups. The taste score was found to be significantly higher in the group treated with yeast for 3 h and it was evaluated by the panelists as the most delicious squid among all of the groups. As a result of the study, it was determined that the chemical, physical, and sensory properties of squid could be improved by marinating with yeast, milk, and mineral water for different durations.

## Graphical abstract

The results of the analyses showed that different treatments and their durations affected the dry matter, ash content, lipid, and crude protein of the squid. Different marinade treatments did not affect the tissue parameters of the squid but hardness, springiness, cohesiveness, and gumminess properties statistically differed among marination times. The taste score was found to be significantly higher in the group treated with yeast for 3 h and it was evaluated by the panelists as the most delicious squid among all of the groups.

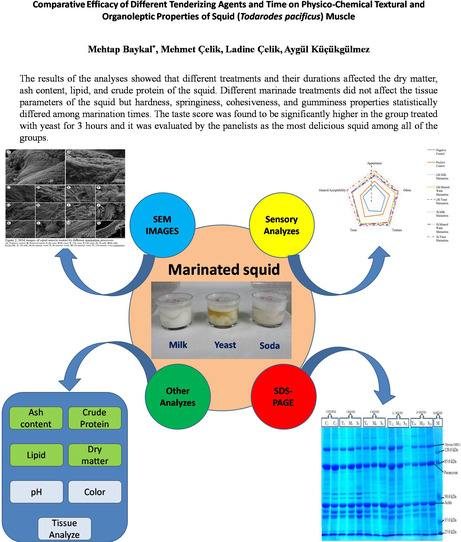

## 1 INTRODUCTION

Seafood has always been among the most important food sources in the human diet due to its difference from other meat groups regarding nutritional value and taste. The high importance of seafood in terms of healthy nutrition as human food stems from the fact that it contains the necessary components for the body at the desired level. Along with its easy digestibility, seafood meat also contains high protein and fat content. It is a food source that is rich in polyunsaturated fatty acids (i.e., ω3 and ω6) (Kaya et al., 2004). Studies have shown that ω3 fatty acids have ameliorating and protective effects against many diseases when consumed regularly. It has been reported that its regular consumption exhibits protective effects against cardiovascular diseases, ameliorating effects against hypertension, diabetes, migraine, depression, cancer, and rheumatic calcification as well as lung, stomach, intestine, and dermatological disorders, and has a positive impact on maternal–infant health (Al‐Hajj et al., 2014; Arıman & Yandı, 2006; Kaya et al., 2004). The fact that seafood contains a variety of minerals and vitamins such as iron, calcium, potassium, selenium, zinc, niacin, and folic acid; vitamins A, D, E, K, B3, B6, and B12; iodine, fluorine, phosphorus, and vanadium sulfide further increases its importance (Kaya et al., 2004; Shi et al., 2015).

Among the seafood products that are so important regarding their nutritional value, mollusks and especially squids among mollusks step forward due to their high biological and economic value. Squids, as an important and delicious food source, are found in all seas and oceans across the globe, especially in the countries surrounding the Mediterranean and its east. Squids are known to represent an important resource with their high productivity and high growth rates. The total amount of squid caught worldwide was estimated to be 2.104.000 tons/year (FAO, 2022). Squid is known to exert a positive impact on human health due to its high‐protein and low‐fat content. Squid, a considerably healthy and dietetic food due to its high content of vitamins and minerals, has various cooking and presentation styles specific to countries. The most substantial problem of squid meat is its intrinsic hard structure. This hardness originates from the muscle structure, mantle, and connective tissue. The high content of insoluble myostromin in squid shell reveals a texture that makes the meat tough and difficult to chew (Grygier et al., 2020). Therefore, the processing method is considerably important (Melendo et al., 1997). Collignan and Montet (1998) reported that cooking for more than 1 min at 100°C gelatinized the collagen and made the squid meat more tender, but cooking for more than 5 min led to excessive hardening. Hu et al. (2014) achieved the desired level of tenderization by employing ultrasound waves of varying frequencies. However, it has been stated that it is a practice that is not accepted by consumers due to its safety.

Various enzymatic methods (i.e., endogenous and exogenous enzymes) are used to tenderize the squid meat (Gökoğlu et al., 2017; Ketnawa & Rawdkuen, 2011). In this way, it is aimed to have the desired taste and texture in squid meat. Typically, pH, time, and temperature are the factors that influence the activity of endogenous enzymes. Another application employed in squid meat marination is herbal (papain, bromelain, ficin) enzymes (Chacko, 2016; Mehrabani et al., 2022; Azmi et al., 2023). All these methods result in tenderization of meat that reduces the cooking times, hardness of meat, and chewiness of meat products that can increase meatiness, flavor, and overall palatability (Woinue et al., 2020). Squid, which is preferred in menus containing seafood and has a hard meat structure in terms of chewiness, must be marinated before consumption. Therefore, in this study, it was aimed to evaluate the properties (chemical, physical, and sensory) that different marination materials (yeast, milk, and mineral water) add to the structure and flavor of squid (*Todarodes pacificus*) meat.

## 2 MATERIALS AND METHODS

### 2.1 Preparation of samples

In the present study, Japanese common squid (*Todarodes pacificus*) meats were used. Two control groups were assigned. In the negative control group, the thawed squid rings were used immediately, whereas, in the positive control group, they were treated with 10 g dry carbonate and 10 g dry salt for presoftening for 2–3 min. This was applied before the marination process of all groups. Three experimental groups were assigned as yeast, milk (1.7 g/100 mL of fat content), and mineral water (mineral content: calcium: 170, potassium: 40, magnesium: 72, sodium: 186, bicarbonate: 1125, sulfate: 25.01, and chloride: 63.87 mg/L), with a squid meat/marinade ratio of 1/1.5. All marination processes were carried out at +4°C. Marinated samples were preserved in glass jars. Samples were taken at the 3rd, 6th, 12th, and 24th hours of the marination process and analyzed. The study was conducted in two replications and each analysis was performed in three parallels.

### 2.2 Proximate analysis

Moisture and ash analyses were performed according to the method of AOAC (1990) after the homogenization of the samples. Nitrogen content was converted to calculate the crude protein content as per Kjeldahl's method (AOAC, 1990). Lipid content was assayed as per Bligh and Dyer (1959); and pH analyses were done according to the method of Lima Dos Santos et al. (1981).

### 2.3 Sodium dodecyl sulfate–polyacrylamide gel electrophoresis (SDS‐PAGE) analysis

Protein profiles were compared in sodium dodecyl sulfate–polyacrylamide gel electrophoresis (SDS‐PAGE) according to Laemmli (1970)'s method. SDS‐PAGE consists of two separate layers that have two different densities. The upper stacking gel allows proteins to clump together and enter the separation gel together. The separation gel, on the other hand, is denser than the stacking gel and it is the layer where the bands are formed in the process of proteins being separated from each other based on their molecular weight.

### 2.4 Texture analysis

Tissue analyses were made with modifications to the methods of Salvador et al. (2002) and Deng, Luo, et al. (2014) using a texture analyzer instrument (TA‐XT2I) equipped with a 36 mm probe. Hardness, springiness, cohesiveness, and gumminess values were read and recorded. Samples having approximately 4 cm diameter were taken from different parts of each squid meat for the measurements.

### 2.5 Scanning electron microscopy (SEM) analysis

Samples were hydrated in distilled water and cut from gels as cubes with a side length of 2 × 3 mm for microscopic examination. They were then fixed in 2% osmium tetroxide (OsO_4_) for 2 h, rinsed with distilled water, and dehydrated with a gradual acetone series. Samples were dried at the critical point by using liquid carbon dioxide as the exchange medium, mounted on aluminum rods, and coated with platinum. Coated samples were then examined under a Zeiss Supra 55 (FESEM, Germany) scanning electron microscope.

### 2.6 Colorimetric analysis

Calder's (2003) approach was used to take colorimetric measurements. A color analyzer instrument (Hunter Associates Laboratory, Inc.,) was used to determine the color of the samples. For the analysis, the sensor was standardized with white and black tiles.

The values of L*, a*, and b* were recorded. The color of the squid meat samples was measured in three distinct parts and the chroma, hue, and whiteness values were determined using the following equations:

(1) $$\text{Chroma}={\left(a^{*2}+b^{*2}\right)}^{1/2}$$

(2) $$\mathrm{Hue}=\text{Arctan}\left(b^{*}/a^{*}\right)$$

(3) $$\text{Whiteness}=100-{\left[{\left(100-L^{*}\right)}^{2}+a^{*2}+b^{*2}\right]}^{1/2}$$

The chroma value shows the distance from the neutral axis and is a measure of saturation. Angles ranging from 0 to 360 degrees are used to represent the hue value.

### 2.7 Sensory evaluation

For the sensory evaluation of the squid meat samples, ten panelists were recruited from the faculty members of the fisheries faculty who were familiar with seafood products. The sensory descriptors were established in collaboration with the panelists during training sessions and prior to tasting. Five criteria were generated: appearance, odor, texture, taste, and overall acceptance. A 10‐point hedonic scale was used to assess the sensory experience. On the scale, scores between 9.0 and 10.0 indicated ‘extremely good’, scores between 7 and 8 indicated ‘good’, scores between 5 and 6 indicated ‘acceptable’, and the limit of the acceptance was 3.9.

### 2.8 Statistical analysis

Data were analyzed by ANOVA as repeated measures using the MIXED procedure of SAS. The least squares means were estimated by a restricted maximum likelihood method. The statistical model included the effects of treatment (T), time (H), and interaction between treatment and time (TxH). The first‐order autoregressive was used as a covariance structure. Time was used in the REPEATED statement with squid within treatment as the random term, where significant treatment means were separated using the PDIFF option of SAS (Littell et al., 2006).

## 3 RESULTS AND DISCUSSION

The chemical and physical properties of the raw squid meat (negative and positive control) are given in Table 1. No treatment was applied to the negative control groups whereas the positive control group was subjected to dry salt and carbonate pretreatments.

**TABLE 1:** Chemical and physical properties of squid meat.

	Negative control	Positive control
Dry matter (%)	13.97 ± 0.04	13.87 ± 0.30
Crude ash (%)	1.69 ± 0.40	1.25 ± 0.05
Lipid (%)	0.39 ± 0.03	0.60 ± 0.03
Protein (%)	9.90 ± 0.11	10.14 ± 2.17
pH	8.89 ± 0.01	8.55 ± 0.02
L*	72.87 ± 0.60	64.89 ± 0.42
a*	−3.03 ± 0.19	−3.30 ± 014
b*	6.49 ± 0.99	3.97 ± 1.19
Chroma	7.17 ± 0.91	5.20 ± 0.97
Hue	−1.12 ± 0.06	−0.86 ± 0.13
Whiteness	71.93 ± 0.70	64.49 ± 0.43
Hardness (*N*)	364.67 ± 84.73	287.23 ± 15.11
Springiness (mm)	0.905 ± 0.04	0.975 ± 0.00
Cohesiveness	0.705 ± 0.07	0.503 ± 0.04
Gumminess (*N*)	259.81 ± 78.78	140.54 ± 12.84

*Note*: ±Standard error.

Dry matter, ash, lipid, and protein rates of untreated squid meat were found to be 13.97%, 1.69%, 0.39%, and 9.90%, respectively. There is a great number of studies with regard to the nutritional composition of fresh squid meat. It has been reported that the moisture content of squid meat varies between 75% and 84% (Çoban & Patır, 2010; Gökoğlu et al., 1999; Sikorski & Kolodziejska, 1986). Similar to the present findings, Zlatanos et al. (2006) found that the ash content in the squid meat obtained from the Mediterranean was 1.5% while Sikorski and Kolodziejska (1986) reported a range of 0.9%–1.9%. Furthermore, Sikorski and Kolodziejska (1986) showed that squid meat contains 13%–22% protein.

### 3.1 Proximate composition

In the present study, changes occurred in the nutritional composition of the squid meat during marination for 3, 6, 12, and 24 h with milk, mineral water, and yeast are given in Table 2.

**TABLE 2:** Proximate compositions and pH changes of squid muscle treated by different marination processes.

		Time (h)	Dry matter (%)	Crude ash (%)	Protein (%)	Lipid (%)	pH
Treatment	Yeast	3	13.8^ab^	1.38^a^	10.7^ab^	0.63^d^	8.23^cd^
		6	14.4^ab^	1.06^ef^	7.5^d^	1.07^bc^	7.46^e^
		12	14.14^ab^	1.00^f^	11.0^a^	1.26^b^	7.71^e^
		24	13.6^abc^	1.00^f^	9.8^c^	1.24^b^	7.74^e^
	Milk	3	13.5^abc^	1.38^a^	5.1^f^	0.66^d^	8.45^ab^
		6	13.8^abc^	1.13^cde^	9.9^c^	1.19^bc^	8.28^bc^
		12	14.0^ab^	1.18^bcd^	10.2^bc^	1.20^bc^	8.30^bc^
		24	14.9^a^	1.12^de^	9.9^c^	1.24^b^	8.51^a^
	Mineral water	3	13.7^abc^	1.21^bc^	5.8^e^	0.96^c^	8.14^cd^
		6	14.1^abb^	1.22^bc^	10.4^abc^	1.04^bc^	8.07^d^
		12	12.1^c^	1.27^b^	10.2^bc^	1.15^bc^	8.52^a^
		24	13.1^bc^	1.05^ef^	10.1^bc^	1.90^a^	8.29^bc^
SEM			0.33	0.018	0.14	0.047	0.035
P		T	0.001	0.001	0.001	0.001	0.001
		H	0.001	0.001	0.001	0.001	0.001
		T x H	0.001	0.001	0.001	0.001	0.001

*Note*: Values followed by the different superscript letters within each column are significantly different.

Abbreviations: H, Time (Hour); SEM, Standard error of the means; T, Treatment; Tx, indicates the statistical differences of treatment and time interaction.

There was an interaction effect between the dry matter levels of the samples and different marination applications and times. It was found that the dry matter level was high in the yeast and mineral water groups at the 3rd hour of the marination. It was observed that the dry matter level in all three treatment groups increased at the 6th hour of the marination and decreased in yeast and mineral water treatment groups at the 12th hour, but increased in the milk treatment group. At the 24th hour of the study, an increase was observed in the milk and mineral water groups while the dry matter level in the yeast treatment group continued to decrease. It has been reported that the water content of the meat depends on its ability to absorb the marinade (Yusop et al., 2010) and the water retention capacity varies based on the composition of the marinade (Latif, 2010).

According to the results of the present study, the dry matter level of the meat varied depending on the treatment sources. Since the dry matter levels of yeast and milk are 8% and 12.6%, respectively (Atalay et al., 2019; Gürsoy, 2010), it was observed that the dry matter levels of the meats treated with yeast and milk increased compared to the mineral water group. Consequently, this increase in the dry matter level can be attributed to the high quantity of dry matter in yeast and milk marinades, and possibly even to the high ability of squid meat to absorb the yeast and milk marinades.

It was observed that there was a relation between the ash levels of the squid meat, different marination applications (yeast, milk, and mineral water), and their durations (3, 6, 12, and 24 h) (Table 2). The ash levels of the yeast and milk groups were high at the 3rd hour of the marination. At the 6th hour, a significant decrease was determined in the yeast and milk groups, and this decrease continued in the yeast group at the 12th and 24th hours. However, the ash contents of the groups marinated with milk and mineral water increased at the 12th hour. At the 24th hour, the ash level in all three treatment groups decreased due to the prolongation of the marination time. In the present study, it was found that the ash levels in the squid meats marinated with different treatments for different durations differ from the findings of previous studies (Çelik et al., 2002; Gökoğlu et al., 1999). The salting processes of the squid meat samples before marination may have also been effective in this difference.

As seen in Table 2, the interaction between treatment and time influenced the lipid level of the squid meat. It was observed that all treatments increased the lipid concentration depending on the increase in the marination duration and there was a further significant increase in the mineral water group at the 24th hour. In the present work, the lipid concentrations increased, especially in the mineral water treatment at the 24th hour, in parallel to the extended marination durations. It is thought that this increase in the mineral water group was due to the proportional increase in the lipid content as a result of the decreased dry matter level (caused by the osmoregulation in the tissue).

Regarding the treatment and time interaction, the crude protein was determined to be the highest in the yeast treatment group and the lowest in the milk treatment group at the 3rd hour. At the 6th hour, it was observed that the crude protein in the yeast group decreased while it increased in the milk and mineral water groups. It was found that the crude protein in the yeast group increased at the 12th hour of marination and partially decreased at the 24th hour. Moreover, all treatment groups displayed similar protein levels at the 24th hour. According to these findings, it was determined that marination for 3 h with yeast and 6 hours with both milk and mineral water is adequate for high crude protein levels in squid meat. Research suggests that the diversity of myofibrillar proteins and the intense autolysis formation in squid meat cause difficulties in squid processing (Sánchez‐Alonso et al., 2007). Processing of squid in alkaline and acidic conditions improves the protein level of squid (Palafox et al., 2009).

It was observed that yeast, milk, and mineral water treatments affected the pH level of the squid meat (*p* < .01). The highest pH was determined in the milk group (8.38) followed by the mineral water group (8.25) while the lowest pH was observed in the yeast group as 7.85 (Table 2). At the 3rd hour of the marination, the highest pH was observed in the milk group. There was a decrease in all treatments at the 6th hour whilst the sharpest decrease was in the yeast group. At the 12th hour of the marination, an increase was observed especially in mineral water and yeast groups. Furthermore, the pH of the mineral water group decreased at the 24th hour whereas that of the milk group increased. However, previous studies have shown that the pH of squid meat varies between 6.3 and 6.8 (Çelik et al., 2002; Çoban & Patır, 2010; Gökoğlu et al., 1999). On the other hand, several researchers suggest that the acidity of the marinated meat depends on the pH level of the marinade as in the present study (Özden & Baygar, 2003).

Several enzymatic and nonenzymatic methods are employed to tenderize squid meat and mainly either endogenous or exogenous enzymes are used. The pH of the meat has an impact on the activity of the endogenous enzymes (Collignan & Montet, 1998). The pH levels of yeast, milk, and mineral water used in the present study are 4.5, 6.5, and 6.7, respectively (Atalay et al., 2019; Gürsoy, 2010). It was determined that the pH level of the yeast group decreased due to the pH of the yeast. Contrary to these findings, Guldas and Hecer (2012) found that the treatment of squid meat with different solutions does not affect pH and acidity. The findings of the current study, on the other hand, suggest that the difference in the pH levels of the yeast, milk, and mineral water led to changes in the pH of the squid meat. Melendo et al. (1997) reported that the decrease in pH does not affect the hardness of the meat. Similarly, Sikorski and Kolodziejska (1986) and Kolodziejska et al. (1987) showed that the pH level of the squid meat as a consequence of different treatments does not affect the meat quality. However, the fact that the pH levels of different treatments were found to be close to neutral pH in the present work may be considered as a favorable approach to the meat quality.

### 3.2 SDS‐PAGE

**FIGURE 1:**
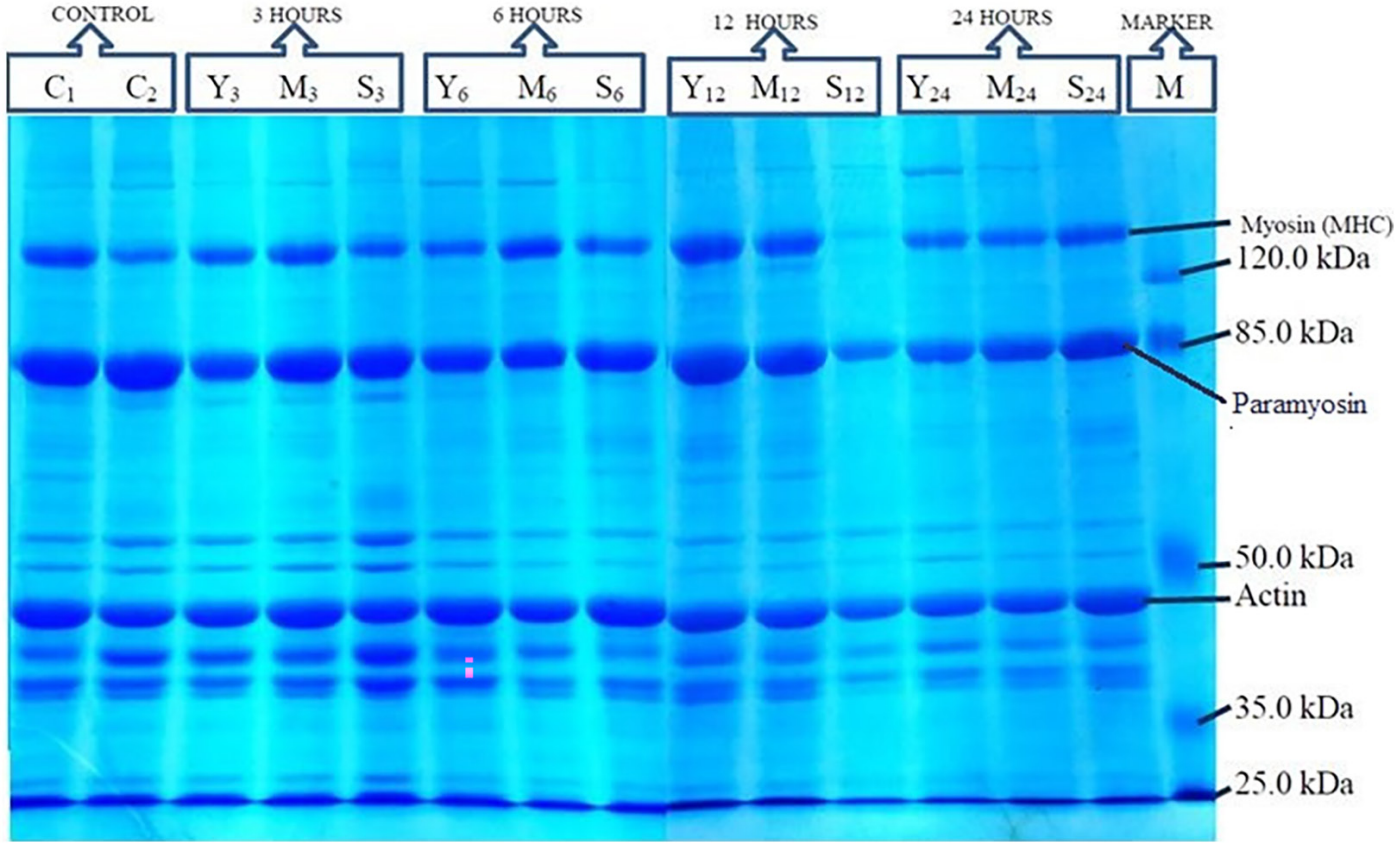
SDS‐PAGE analysis of proteins in squid muscle treated by different marination processes (C1:Negative control, C2: Positive control, Y: Yeast, M: Milk, S: Mineral water).

Figure 1 shows the electrophoretic changes in the squid meat proteins at different times of the marination with different products.

The protein changes that occurred during the marination of the squid meat with yeast, milk, and mineral water are given in Figure 1. As seen in the figure, myosin heavy chain, paramyosin, actin, and minor bands were present in the gel. It was observed that there was a slight decrease in the band intensities according to different treatments and their durations. However, this did not show substantial changes in intense bands such as MHC, AC, and P. Similar to the results of the present study, Ramírez‐Suarez et al. (2008) reported a slight decrease in myosin band intensity, a slight increase in paramyosin band intensity, and the presence of a 153 kDa contaminant band in jumbo squid (*Dosidicus gigas*) meat stored at 0°C for 15 days. Rodger et al. (1984) found a number of changes in electrophoretic patterns after activating an endogenous alkaline protease. Collignan and Montet (1998) demonstrated by electrophoretic analysis that tenderization of squid mantle tissue occurs through proteolysis, and the size of the proteolytic products changed significantly, especially at low molecular weights. Sakai and Matsumoto (1981) reported that the squid (*Ommastrephes sloani pacificus*) shows autolytic activity at acidic pH. Likewise, Konno and Fukazawa (1993) found that *Todarodes pacificus* has high autolytic activity through myosin hydrolysis at pH 7 at 40°C.

### 3.3 Texture profile

The results of the tissue changes that occurred during the marination of the squid meat with milk, mineral water, and yeast at different periods in the present study are presented in Table 3.

**TABLE 3:** Texture profiles of squid muscle treated by different marination processes.

		Time (h)	Hardness (*N*)	Springiness (mm)	Cohesiveness	Gumminess (*N*)
Treatment	Yeast	3	331.8^a^	0.856^ab^	0.640^a^	210.9^ab^
		6	359.7^a^	0.888^a^	0.633^a^	233.2^ab^
		12	279.2^ab^	0.850^ab^	0.566^ab^	174.5^ab^
		24	298.3^ab^	0.912^a^	0.552^ab^	142.0^ab^
	Milk	3	267.8^ab^	0.881^ab^	0.537^ab^	151.4^ab^
		6	260.7^ab^	0.901^a^	0.560^ab^	148.4^ab^
		12	249.3^b^	0.875^ab^	0.454^b^	113.3^b^
		24	343.4^a^	0.839^ab^	0.661^a^	260.8^a^
	Mineral water	3	283.1^ab^	0.864^ab^	0.565^ab^	158.9^ab^
		6	334.6^a^	0.917^a^	0.615^ab^	221.4^ab^
		12	247.3^b^	0.844^ab^	0.542^ab^	136.9^ab^
		24	344.9^a^	0.726^b^	0.671^a^	233.1^ab^
SEM			29.63	0.03	0.04	27.53
P		T	0.242	0.233	0.150	0.511
		H	0.035	0.048	0.014	0.02
		T x H	0.280	0.065	0.065	0.007

*Note*: Values followed by the different superscript letters within each column are significantly different.

Abbreviations: H, Time (Hour); SEM, Standard error of the means; T, Treatment; TxH, indicates the statistical differences of treatment and time interaction.

Different marination applications did not affect the texture parameters of the squid meat, but the duration of the marination affected the hardness, springiness, cohesiveness, and gumminess properties (*p* ≤ .05) (Table 3). It was found that the hardness, cohesiveness, and gumminess parameters increased at the 6th hour, decreased at the 12th hour, and increased again at the 24th hour of the treatment. Similar to the other tissue parameters, the degree of springiness increased at the 6th hour of the treatment period. It showed a decrease at the 12th hour; however, unlike other parameters, it was found that this decrease continued at the 24th hour.

It is thought that the time‐dependent changes were effective on the lipid, protein, and ash levels of the squid meat. Particularly, endogenous and exogenous enzymes play a role in shaping the structural properties of the tissue. The activity of endogenous enzymes varies depending on the pH, time, and temperature factors (Collignan & Montet, 1998). It was reported that the muscle tenderization achieved by storage in different squid meat varieties occurs through the breakdown or binding of collagen at the tissue level by means of endogenous proteinases (Kagawa et al., 2002). Exogenous proteolytic enzymes provide tenderization of the tissue by partially hydrolyzing the elastin collagen tissue in meat (Melendo et al., 1997). Another group of researchers showed that the textural characteristics of the meat depend on the protein fraction (Olivas et al., 2004).

Cathepsins are the most active proteins in squid meat. Cathepsins are associated with protein denaturation and postmortem deterioration (Ayensa et al., 1999; Hernández‐Andrés et al., 2005). Deng, Wang, et al. (2014) reported that the cathepsin activity decreases as a consequence of thermal application. The high acidity of marination liquids reduces the pH level below the isoelectric point where the swelling and viscous properties of proteins are the lowest. Thus, the structure of the protein is unfolded by electrostatic repulsion and more water can be bound to these regions. A juicy–crispy texture is obtained by increasing the water‐binding capacity of the meat (Gault, 1991).

The phosphate group also increases the pH of the meat, causing proteins to be negatively charged and therefore increases the water retention capacity of the meat (Ergezer & Gökçe, 2004). In the present study, it can also be stated that the acidity decreased partially depending on the duration of the marination with yeast. Moreover, it was observed that all the tissue parameters except the springiness increased with time.

### 3.4 SEM images

**FIGURE 2:**
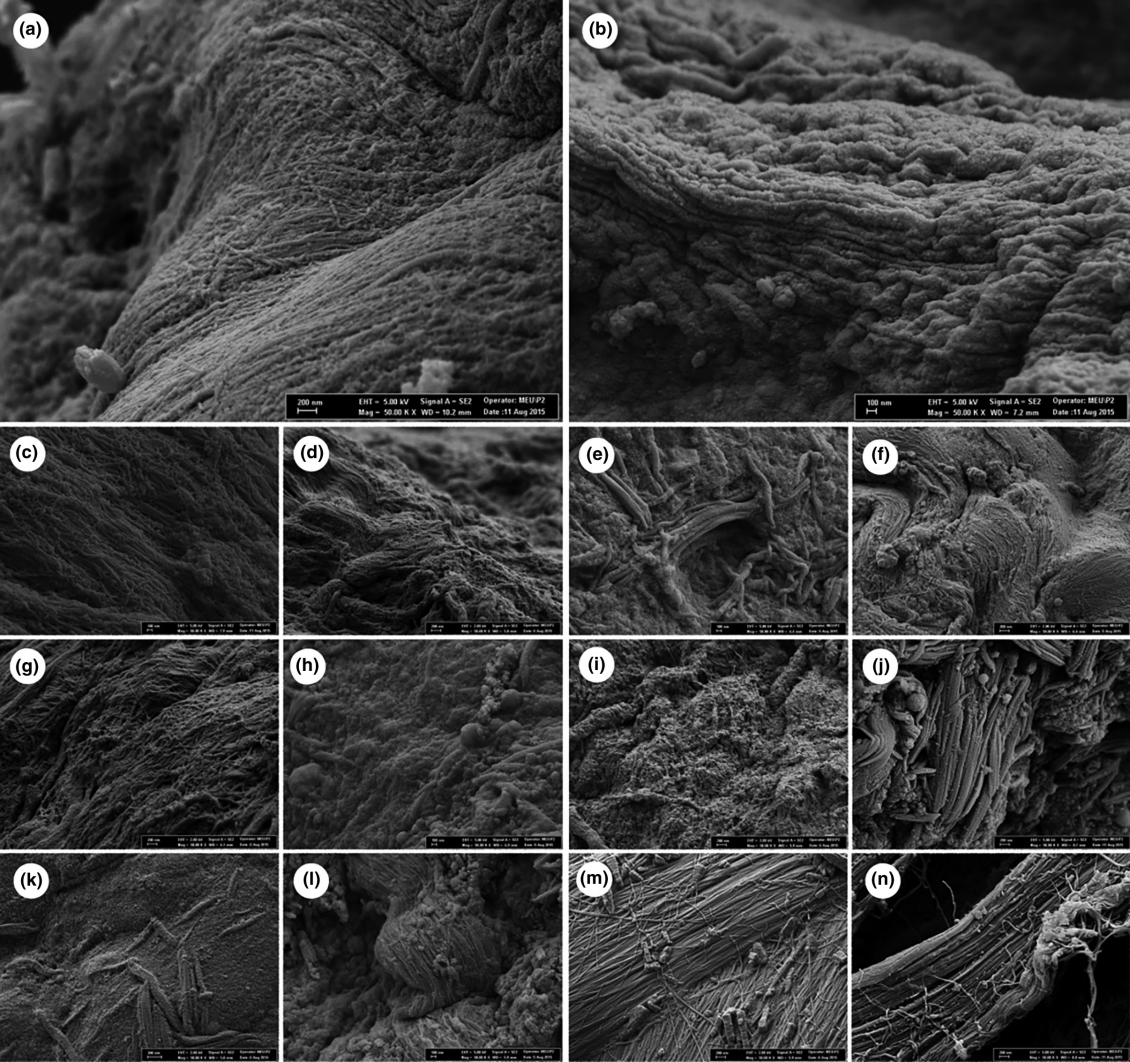
SEM images of squid muscle treated by different marination processes. (a: Negative control, b: Positive Control, c: 3 h yeast, d: 6 h yeast, e: 12 h yeast, f: 24 h yeast, g: 3 h milk, h: 6 h milk, i: 12 h milk, j: 24 h milk, k: 3 h mineral water, l: 6 h mineral water, m: 12 h mineral water, n: 24 h of mineral water marination).

The effect of different marination applications and durations on the muscle fiber arrangement of the squid meat is given in Figure 2. According to the SEM results, the most affected groups by the application and duration were found to be the mineral water group at the 12th and 24th hours, and the milk group at the 24th hour. It was observed that the mineral water group exhibited more fractures at the 24th hour than it did at the 12th hour. The group marinated with the mineral water for 12 and 24 hours displayed soft and fibrous fractures and had more structural fractures than that of the group marinated with milk for 24 h. Fibers in all marinated squid meats were observed to spring significantly more compared to the positive and negative control groups. This confirms the approach that the tissue attained a more tender structure as a result of the applications. Furthermore, there was a significant water loss in the positive control group when compared with the negative control. It was observed that the squid meats treated with milk, mineral water, and yeast interacted with different marination applications and their durations in comparison with both the negative and positive control groups.

The high proteolytic activity of the squid meat necessitates its processing. The endogenous proteases cause physicochemical and structural changes as a result of storing the squid meat at 4°C (Dublán‐García et al., 2006). The texture of the meat mainly depends on the muscle fibers and connective tissue proteins. Muscle fibers are more important than connective tissue proteins. Bugeon et al. (2003) confirmed the relationship between muscle fibers, muscle proteins, and sample hardness. They reported that the springiness increases and hardness decreases as a result of the ultrasonic application compared to the control sample. They concluded that the ultrasonic application is likely to promote the separation of long chains in myofibrils and therefore tenderize the meat tissue. On the other hand, Hu et al. (2014) determined that the control group displayed ring muscle fiber having uniform size, regular shape, thick diameter, and narrow interval. They observed that the cells were in close contact with each other and the intracellular materials appeared to be intact. Furthermore, they determined that, under optimal processing conditions, the ultrasonic application caused severe damage, fractures, and wide gaps in loose muscle fibers. The changes detected in the squid fiber cells indicated that the ultrasonic application damages the muscle fiber structure of the squid meat. The findings of the present study, on the other hand, showed that squid meat can be tenderized depending on the type and duration of the marination.

### 3.5 Color changes

The color of seafood is one of the most important sensory/visual acceptability criteria for consumers. L*, a*, and b* values were measured to monitor the changes in the physical quality parameters of the squid meat during the marination process, and the *whiteness*, *hue*, and *chroma* parameters were calculated from these values. The effects of marination applications, their durations, and their interactions are presented in Table 4.

**TABLE 4:** Color changes of squid muscle treated by different marination processes.

		Time (h)	L*	a*	b*	Whiteness	Hue	Chroma
Treatment	Yeast	3	73.0^ab^	−2.72^bc^	11.2^a^	70.6^bcd^	−1.33^bc^	11.5^a^
		6	74.6^a^	−2.81^bc^	11.0^a^	72.1^abc^	−1.32^abc^	11.4^ab^
		12	74.8^a^	−1.76^a^	12.9^a^	71.6^abc^	−1.44^c^	13.0^a^
		24	69.1^c^	−3.20^bcd^	11.3^a^	66.9^e^	−1.29^abc^	11.8^a^
	Milk	3	73.7^ab^	−3.32^cd^	7.3^b^	72.5^abc^	−1.14^ab^	8.0^c^
		6	74.4^ab^	−2.99^bc^	6.7^b^	73.3^ab^	−1.15^ab^	7.4^c^
		12	74.1^ab^	−2.55^b^	7.1^b^	73.0^abc^	−1.23^abc^	7.6^c^
		24	71.1^bc^	−3.80^d^	8.0^b^	69.7^a^	−1.12^ab^	8.9^c^
	Mineral water	3	75.3^a^	−3.15^bcd^	6.6^b^	74.2^a^	−1.13^ab^	7.3^c^
		6	75.9^a^	−2.56^bc^	6.0^b^	75.0^a^	−1.15^ab^	6.6^c^
		12	68.4^c^	−2.57^bc^	5.9^b^	67.7^de^	−1.12^ab^	6.5^c^
		24	75.4^a^	−2.76^bc^	5.7^b^	74.5^a^	−1.11^a^	6.3^c^
SEM			0.69	0.155	0.59	0.72	0.044	0.53
P		T	0.184	<0.0001	<0.0001	<0.0001	<0.0001	<0.0001
		H	<0.0001	<0.0001	0.553	<0.0001	0.123	0.522
		T x H	<0.0001	<0.0001	0.243	<0.0001	0.689	0.131

*Note*: Values followed by the different superscript letters within each column are significantly different.

Abbreviations: H, Time (Hour); SEM, Standard error of the means; T, Treatment; TxH, indicates the statistical differences of treatment and time interaction.

As a result of different marination applications, the lightness (L*) value was observed to be the highest in the mineral water group at the 3rd hour of the marination. The lightness increased in all treatments at the 6th hour. However, there was a sharp decrease in the mineral water group at the 12th hour. At the 24th hour of the application, it was observed that the lightness increased in the mineral water group again, but it decreased in the yeast and milk groups. Latoch (2020) found that the L* value of the meat decreased as the time of the marination increased. Similarly, in the present study, it was determined that the lightness of the squid meat decreased as the marination duration increased. It was observed that the L* value of the milk group decreased at the 24th hour while the dry matter value increased. This suggests that the water loss may play a role in the loss of lightness as well as that the lightness decreased due to the denaturation of proteins. At the 24th hour of the study, a probable relationship between the dry matter level and lightness was not observed for the yeast and mineral water groups.

It was observed that the yeast and mineral water treatments increased the redness (a*) of the squid meat whereas the milk treatment decreased it. Statistically significant differences were found between the groups in the analysis of variance performed (*p* < .01). In agreement with the findings of the present work, Latoch (2020) stated that the use of dairy products in meat marination decelerates the myoglobin oxidation, increases the thermal stability, and reduces the redness. In the present study, it was determined that the yeast and mineral water applications decreased the a* value, leading to an increase until the 12th hour while a contrary trend was observed at the 24th hour. In light of these data, it can be concluded that the marination process can be performed until the 12th hour. It was previously determined that the tonality of the jumbo squids, that were caught throughout the year, decreased and turned yellow when the squids were stored on ice for 15 days (Ramírez‐Suarez et al., 2008).

Benjakul et al. (2012) emphasized that the color of squid, which was stored in the deep freeze and formed a pink color, can be ameliorated with the use of sodium chloride and an oxidizing agent. As can be seen, the high protein content of the squid meat may undergo oxidation depending on the catch periods and processes applied. In the present study, it was observed that the use of milk in the marination decelerated the possible oxidation. It was determined that it is possible to achieve a product that is desirable by the consumer by marinating squid meat with yeast for 12 h.

Different treatments significantly affected the yellowness (b*) value of the squid meat (*p* < .01). The highest yellow color was in the yeast group (11.61), followed by the milk (7.27) and mineral water groups (6.05), respectively. The statistical analysis revealed that the duration of the marination with yeast, milk, and mineral water did not affect the b* value (*p* > .05). Guldas and Hecer (2012) reported that a yellowish color was observed when the squid meat was marinated with yeast extract. It has been shown by Kong et al. (2007) that proteins are denatured and pigments are oxidized by thermal processing. In the present work, it was observed that the marination with yeast was effective in the formation of the yellow color. It is likely that the activation of the yeast with the increase in the temperature influenced the increase of the b* value.

The marination with milk and mineral water affected the whiteness of the meat (*p* < .001). The highest whiteness values was determined in the milk (72.12) and mineral water (72.88) groups followed by the yeast (70.30) group. It was found that the duration of the marination with yeast, milk, and mineral water caused statistically significant differences between the groups. The whiteness increased at the 3rd and 6th hours and decreased at the 12th and 24th hours of the marination (*p* < .01). Whiteness is affected by the changes in protein modifications as a result of cooking (Torres‐Arreola et al., 2017). It has been reported that the protein solubility and gel production of the squid are high and squid may even be a source of colorless protein production (De La Fuente‐Betancourt et al., 2009).

It was determined that the treatment of the squid meat with different marinades caused statistical differences between the groups. The treatment with yeast decreased the hue value of the meat, whereas treatment with milk and mineral water increased it (*p* < .01). It was found that the duration of the marination applied to the squid meat did not statistically affect the hue value (*p* > .05). Chroma is an indicator of saturation. It shows the concentration and denaturation of myoglobin, which plays an iron and oxygen‐binding role in the muscle tissue (Sánchez del Pulgar et al., 2012). The higher this parameter, the higher the concentration of myoglobin and the lower the denatured myoglobin content is (Ledward, 1992).

In the present study, the chroma was the highest in the yeast treatment and the lowest in the mineral water group. Latoch (2020) found that the use of dairy products in the marination of meat does not affect the chroma value; however, the chroma value was lower in the low‐temperature application compared to the high‐temperature cooking. Accordingly, it can be concluded that the mineral water group decelerated the oxidation of myoglobin, while the yeast group accelerated it.

### 3.6 Sensorial properties

**FIGURE 3:**
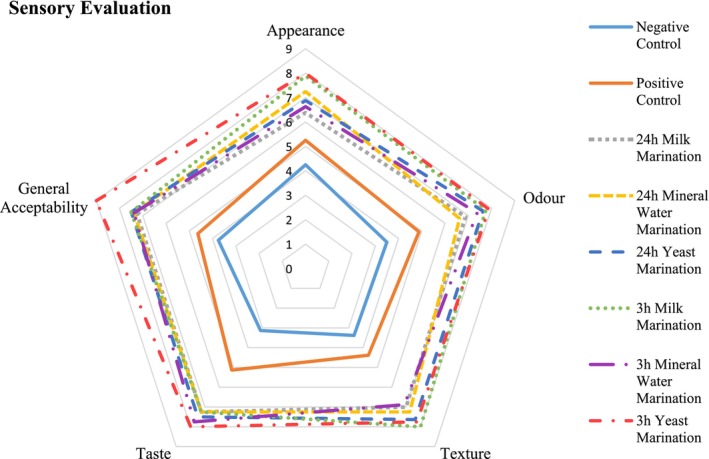
Sensory properties of squid muscle treated by different marination processes.

The results of the effects of different marination applications on the sensory changes of the squid meat are given in Figure 3.

In terms of appearance, the most favored group by the panelists was the yeast marination for 3 h. The score for the appearance was found to be high in the mineral water marination for 24 h (7.25) and milk marination for 3 h (7.88). It was evaluated that the lowest scores were in the negative and positive control groups (4.25 and 5.25, respectively). When the odor parameter of the sensory evaluation was examined, it was observed that the most favorable results were in the treatments of yeast and milk for 3 h. Treatments with yeast for 24 h and with mineral water for 3 h were scored as “good” while this level was lower in the control groups.

When the sensory texture scoring of the squid meat was examined, it was determined that there were significant differences between the treatment groups and the control groups. All of the groups that were treated with marinades were highly favored by the panelists. In particular, the group treated with milk for 3 h received the highest score. With regard to the taste parameter of the sensory evaluation, it was observed that all marinated squid meats were found to be delicious by the panelists. The highest score for taste was in the group treated with yeast for 3 h. Although it was determined that the control groups were lower than the marinated groups, the positive control was found to be higher than the negative control group.

The overall acceptance score was determined to be the highest in the group treated with yeast for 3 h while the control groups had lower scores. The scores of other marinated groups were found to be similar to each other. Similar to the present study, Melendo et al. (1997) concluded that the marination time should be maintained at 0.5 or 2 h. It was reported that the processing of the squid meat in alkaline and acidic environments improves the taste and odor (Palafox et al., 2009). As in previous studies, different marinades used in the tenderization of the squid meat were found to be successful in the present work. When the sensory evaluation data were interpreted, it was concluded that the marinated groups were favored more and received higher overall acceptance scores in comparison with the control groups. It was found that the sensory evaluation scores decreased in parallel to the increase in the tenderization duration.

## 4 CONCLUSIONS

In the present study, it was investigated the effects of different tenderizing agents and time on physicochemical textural and sensorial properties of squid (*Todarodes pacificus*) meat. Although the most effective marinade of squid is with yeast, it has also been shown that it can be marinated with milk or mineral water. However, it has been determined that the most suitable time for marination in all methods is 3 h. It should be noted that meat tenderizing using milk, mineral water, and yeast reduces the use of chemical compounds. The results presented herein demonstrated that yeast, milk, and mineral water improved squid muscle tenderness and can be utilized to ensure a more desirable squid product.

## AUTHOR CONTRIBUTIONS

**Mehtap BAYKAL:** Conceptualization (equal); formal analysis (lead); methodology (supporting); resources (lead); writing – original draft (lead). **Mehmet Celik:** Conceptualization (equal); data curation (equal); methodology (supporting); supervision (lead); writing – review and editing (supporting). **Ladine Celik:** Conceptualization (supporting); data curation (supporting); methodology (equal); resources (supporting); writing – review and editing (equal). **Aygul Kucukgulmez:** Conceptualization (equal); methodology (equal); resources (supporting); supervision (supporting); writing – review and editing (equal).

## FUNDING INFORMATION

The publication fee of the study is supported by TUBITAK (TUB1).

## CONFLICT OF INTEREST STATEMENT

The author declares no conflict of interest with respect to the research, authorship, and/or publication of this article.

## Data Availability

All data generated in this study are found in this manuscript.
